# CO_2_ Valorization and Its Subsequent Valorization

**DOI:** 10.3390/molecules26020500

**Published:** 2021-01-19

**Authors:** Juan Antonio Cecilia, Daniel Ballesteros Plata, Enrique Vilarrasa García

**Affiliations:** 1 Department of Inorganic Chemistry, Crystallography and Mineralogy, Campus de Teatinos, Universidad de Málaga, 29071 Málaga, Spain; daniel.ballesteros@uma.es; 2 Grupo de Pesquisa em Separações por Adsorção, Department of Chemical Engineering, Campus do Pici, Universidade Federal do Ceará, Fortaleza C 60455760, Brazil; enrique@gpsa.ufc.br

After the industrial revolution, the increase in the world population and the consumption of fossil fuels has led to an increase in anthropogenic CO_2_ emissions. These emissions are directly related to a progressive increase in the temperature on the surface of Earth causing global warming, which is seriously affecting the environment and the living beings of the planet. The Intergovernmental Panel on Climate Change (IPCC) has published several reports which indicate the effects of global warming as well as the importance of minimizing anthropogenic CO_2_ emissions to prevent more severe damage on Earth in order to achieve a cycle of zero-CO_2_ emissions in the year 2050 [1]. Considering the severe effects of global warming, both governments and many scientists are making efforts to develop various strategies to reduce CO_2_ emissions into the atmosphere.

Nowadays, various energy sources have been proposed to replace traditional fossil fuels. However, energy demands are so high that it is impossible to substitute fossil fuels in the short term. Considering these premises, the most mature strategy is focused on the design of more efficient processes where CO_2_ emissions can be minimized and, on the other hand, CO_2_ sequestration and its subsequent valorization to obtain high added value chemicals.

The task of reducing CO_2_ emissions is very complex, but at the same time very exciting, since more efficient processes and technologies to capture CO_2_ require offsetting emissions between 100 and 1000 Gt throughout this century [1].

This special issue is focused on highlighting new approaches to capturing CO_2_ from industrial sources such as electricity-generated power, refineries, steel or cement among others, as well as from the ambient air [2]. Once the CO_2_ has been captured, the next challenge is to valorize this compound obtained in large proportions to give rise to other compounds that may be of great commercial interest. In this sense, several catalytic applications have been detailed in the literature such as the synthesis of fuels, drugs or building block molecules to obtain a wide range of valuable products. In addition, CO_2_ can be also employed in photocatalytic process or artificial photosynthesis and in the polymers field [3,4].

In the carbon capture and storage (CCS) process, it has been reported that between 50–90% of the global cost is attributed to CO_2_ capture [5] so one of the main efforts for the scientific community is associated to the development of efficient processes for CO_2_ capture. Among technologies proposed for the CO_2_ capture, it can be highlighted cryogenic distillation, membrane purification, absorption and adsorption [5]. Both cryogenic distillation and membrane purification appear highly efficient in short-scale; however, there are limitations for the larger-scale, as well as diluted CO_2_-flows. In addition, the cost of these processes is quite expensive for the large amounts of CO_2_ that should be retained [6]. The most mature technology used to retain CO_2_ molecules is absorption with amines or chilled ammonia obtaining excellent results, although a strong drawback is observed related to the high corrosivity and the costs required for the regeneration of the amines [7].

The design of materials with the appropriate physicochemical properties seems to be the most sustainable technology for CO_2_ capture. There is a wide range of adsorbents with the potential to capture CO_2_. However, it is necessary to highlight that the cost of CO_2_ capture can reach 90% of the global cost of the CCS process so efficient materials for CO_2_ capture are required, but at the same time these materials must be sustainable from an economic point of view. In this sense, alkaline and mainly alkaline-earth oxides have shown excellent behavior in CO_2_ capture although these materials display a serious drawback related to their regeneration because of the strong interaction of these oxides with the CO_2_ molecules [8]. On the other hand, a wide range of porous materials have been designed as molecular sieves and then tested in CO_2_ adsorption processes. In the last decade, both metal organic frameworks (MOFs) and graphene organic frameworks (GOFs) have been developed to retain molecules [9,10]. These materials display 3D ordered structures with narrow and homogenous pore size in such a way that these porous materials can trap CO_2_ molecules in their structures. The main drawbacks of these materials are related to the high cost of synthesizing adsorbents in large-scale and the relatively low thermal stability of MOFs and GOFs. Other materials with small and narrow pore diameter are zeolites and activated carbons. Both adsorbents have been synthesized on larger scale for several adsorption and catalysis processes, attaining high CO_2_ adsorption capacity [11,12]. The design of porous silica with homogeneous and narrow pore distribution has also emerged in the last decades as adsorbent or catalytic support. In the same way, these porous silicas have been selected for CO_2_ capture although the adsorption capacity was lower than that observed for the adsorbents indicated previously [13,14]. Clay minerals are other porous materials that have been tested to capture CO_2_. These materials have aroused great interest due to their low cost and high availability [15].

In all cases, these adsorbents can improve their adsorption capacity by the incorporation of amine-species. The main strategies reported in the literature are grafting with amine-alkoxisilanes [16] or the impregnation of amine-rich polymers [13,14]. In both cases, the CO_2_ adsorption capacity increases because of the existence of chemical interactions via zwitterion forming carbamate in dry conditions or bicarbonate under wet conditions [5].

Nowadays, there are three approaches to CO_2_ capture from the combustion of fossil fuels (precombustion, oxyfuel combustion, and postcombustion) [17,18]. Postcombustion CO_2_ capture is well known from the 1970s as a potential economic source of CO_2_ for enhanced oil recovery operations so this process is ready today while both precombustion and oxyfuel combustion are still in development [17,18].

The main efforts in the CCS process must be focused on the development of innovative technologies to increase the efficiency of the systems. In this sense, several parameters, such as the influence of the solvent, configuration of absorption and stripping columns, operating conditions of columns, percentage of CO_2_ avoided, captured CO_2_ purity and the regeneration steps, must be considered [3].

Once CO_2_ is captured, the next challenge is its valorization into high-added value products. Generally, it has been reported that CO_2_ can be valorized through physical utilization or chemical valorization to form valuables products [19].

As itself, CO_2_ can be directly used to carbonate drinks, produce dry ice, refrigerant, welding medium or fire extinguishers, among others. However, these applications are limited so the effect on the mitigation of CO_2_ emissions is negligible [3]. Pure or dissolved CO_2_ can also be employed in enhanced oil recovery, enhanced gas recovery or enhanced geothermal systems.

When CO_2_ is valorized from physical utilization, these molecules remain unaltered in their form pure or in solution without any chemical reaction.

Several authors pointed out that the injection of CO_2_ or CH_4_/CO_2_ improves oil recovery in homogeneous hydrocarbon reservoirs, so it is a promising alternative for the exploitation of oil reserves [20].

The integration of a methanol plant with enhanced gas recovery and geo-sequestration reported that CO_2_ capture sequestration and utilization has the potential to digest a natural fed gas with a maximum CO_2_ mole fraction of 0.232 [21]. About 83.8% of the CO_2_ captured was employed in enhanced gas recovery while 16.2% was employed in the production of fertilizers [22].

CO_2_ has been also used as geo-fluid for enhanced geothermal systems to obtain geothermal energy [23]. On the other hand, CO_2_ can be used as an alternative to synthetic refrigerants in air conditioning leading to highly efficient systems where fuel consumption diminished [24].

CO_2_ can also be used as a feedstock in the synthesis of valuable chemicals and fuels. The use of CO_2_ as a reactive can reduce resource consumption as well as lower the carbon fingerprint, leading to sustainable chemical processes.

CO_2_ can react with CH_4_ to form syngas through dry-reforming, which can satisfy the demands of valuable chemicals like naphtha or diesel among others [25]. Generally, the components of syngas are H_2_ and CO, although small proportions of CO_2_ and H_2_O can also be observed. The use of CO_2_ in the reforming process allows the combination of steam-methane reforming and dry-methane reforming to reach the optimum H_2_/CO ratio [26]. CO_2_ can also be used in the oxidation of CH_4_, obtaining high proportions of CO and H_2_, which can be used as feed in the reforming of syngas. The excess CO_2_ obtained in the reaction tail can be recycled to the feed again. In the same way, CO_2_ can be converted into CO from the reverse water gas shift reaction using In_2_O_3_ and Ga_2_O_3_ [27].

Methanol can be produced from CO_2_ in the reforming stage (indirectly) and in the methanol reactor (directly) where syngas is converted to methanol [3,28]. CO_2_ utilization in this process was about 0.12 t per metric ton of methanol. The installation of a reverse water gas shift reactor on the recycled stream improves methanol production as well as CO_2_ consumption [29]. As itself, two molecules of methanol can be dehydrated to form dimethyl ether although it can also be formed from syngas directly [30]. The synthesis of methanol can also take place by a photoreduction of CO_2_ and H_2_O [31].

Urea can also be formed from NH_3_ and CO_2_ via carbamide. It has been reported that the synthesis of one ton of urea could consume between 0.735–0.750 tonnes of CO_2_. These authors also indicated that the coproduction of urea and energy can take place simultaneously [3]. There are some studies where CO_2_ coming from syngas of the underground coal gasifier was employed in the formation of carbamide [32], which is considered as an intermediate in the synthesis of the urea. However, the market price of this process is not competitive yet [33].

Dimethyl carbonate has been synthesized from the reaction of phosgene and methanol. However, the high toxicity of phosgene (COCl_2_) has led to the development of alternative synthetic strategies. Nowadays, there are several industrial processes where CO_2_ is used as a reagent to form dimethyl carbonate. Between them, it can be highlighted direct production from methanol and CO_2_, production from CO_2_ and ortho-ester or acetals and production from methanol, CO_2_ and epoxides [34].

Polyurethane is formed by the reaction of CO_2_ and propylene oxide with an alcohol as starter of the reaction and zinc hexacyanocobaltate as catalyst. The synthesis of polyurethane can be one of the most interesting applications to valorize CO_2_ due to the high demand of this product in the market [35]. It has been reported that the amount of CO_2_ employed in the synthesis of polyurethane was 0.3–1.7 kg CO_2_/kg polyurethane [36].

The conversion of syngas to obtain hydrocarbons from the Fischer−Tropsch process has been a challenge throughout the last century. Generally, CO is formed from CO_2_ through the water gas shift reaction. Other authors have also proposed the cofeed of CO-CO_2_ so a small amount of CO_2_ is required to adjust the H_2_/CO ratio [37].

CO_2_ can also be hydrogenated to form CH_4_ via the Sabatier reaction. This reaction is very useful in the purification of syngas as well as in the purification of H_2_ streams in polymer electrolyte fuel cell anodes [38].

Another promising innovative technology for CO_2_ valorization is its use in the chemical-looping dry-reforming process, which consists of three stages (methane reduction, CO_2_ reforming to form CO and oxidation) [39].

Mineralization of CO_2_ to form carbonate species (CO_3_^2-^) is a process where energy is released. This process can be used in cement manufacturing to produce green building materials [40].

Other reagents have been synthesized using CO_2_ as reagent. Between them, ethylene oxide is a chemical formed from ethylene and CO_2_, which is highly used in the chemical industry, mainly in the synthesis of ethylenglycol [3]. In the same way, ethylene and CO_2_ have also been employed to synthesize polyethylene. Supercritical CO_2_ is also used for polybutylacrylate polymerization [3]. On the other hand, CO_2_ can also be employed in the dehydrogenation of propane to propylene [41] or ethylbenzene to styrene [42]. In addition, CO_2_ is also employed to synthesize furan-2,5-dicarboxylic acid, which is considered as a building block with applications in the field of polymers [43]. The CO_2_ molecule can also react with epoxides favoring the ring-expansion or copolymerization reactions [44]. Another catalytic application of CO_2_ is its use in oxidative catalytic activation of small alkanes [45].

In this editorial, the most representative applications have been highlighted. However, the number of applications is uncountable, mainly in fine chemistry or in the field of polymers as well as in the field of biology, since a wide variety of microorganisms can assimilate CO_2_ in anaerobic reactions such as methanation.

The abatement of CO_2_ emissions is an exciting challenge for the scientific community. The objective of solving the problem of global warming can be accompanied by the use and recovery of CO_2_ in value-added compounds. Most of the processes devised to recover CO_2_ are relatively recent in such a way that they are processes that must be optimized to be sustainable and competitive. The greatest efforts should be focused on the integration of processes to save transportation costs. For this purpose, these processes must be supported by environmental policies that promote the capture and valorization of CO_2_.
